# Lymph node metastasis pattern and significance of left gastric artery lymph node dissection in esophagectomy for esophageal cancers

**DOI:** 10.1186/s12957-021-02405-0

**Published:** 2021-10-11

**Authors:** Xiu-Mei Deng, Tian-Yu Zhu, Guo-Jun Wang, Bu-Lang Gao, Jing-Tao Wang, Rui-Xin Li, Yun-Fei Zhang, Heng-Xuan Ding

**Affiliations:** Department of Gastrointestinal Surgery, The First Affiliated Hospital of Zhengzhou University, 1 Jianshe Road, Zhengzhou, 450052 China

**Keywords:** Esophageal squamous carcinomas, Left gastric artery lymph nodes, Lymph nodes labeling, Radical resection, Lymph metastases

## Abstract

**Purpose:**

To investigate the lymph node metastasis pattern and significance of dissection of the left gastric artery lymph nodes in radical en bloc esophagectomy for esophageal squamous carcinomas based on the lymphatic drainage pathway revealed by carbon nanoparticle labeling.

**Materials and methods:**

Patients who underwent en bloc esophagectomy endoscopically were retrospectively enrolled. Carbon nanoparticles were injected in the submucosa of upper thoracic esophagus to label the relevant draining lymph nodes. The clinical data, lymph nodes dissected, surgical technique, and complications were analyzed.

**Results:**

En bloc esophagectomy was successful in all 179 patients. Metastases to the left gastric artery lymph nodes were positive in 42 patients (23.5%) but negative in 137 (76.5%). The left gastric lymph nodes were labeled, whereas no celiac lymph nodes were labeled by carbon nanoparticles. A total of 4652 lymph nodes were resected, with 26 lymph nodes per patient. Seventy-three patients had lymph node metastasis (73/179). Seventeen patients had metastasis to the recurrent laryngeal nerve lymph nodes (9.5%). The metastasis rate of the lower thoracic esophageal cancer to the left gastric artery lymph nodes was 37.0%, significantly greater than that at the middle (15.4%) or upper (6.7%) thoracic segment. The lymph node metastasis rate was significantly (*P* < 0.05) increased with the length of the cancerous lesion, infiltration depth, and poor differentiation. Univariate analysis revealed that the metastasis rate to the left gastric artery lymph nodes was significantly (*P* < 0.05) associated with paraesophageal lymph node metastasis, para-cardial lymph metastasis, and TNM classification. Multivariate analysis indicated that cancer location (odds ratio 8.32, 95% confidence interval 2.12–32.24) was significantly (*P* < 0.05) associated with metastasis to the left gastric artery lymph nodes, with the cancer at the middle and lower thoracic segments significantly more than in the upper thoracic segment.

**Conclusion:**

Certain patterns exist in lymph node metastasis of esophageal cancer, and in radical esophagectomy of esophageal cancers, dissection of the left gastric artery lymph nodes is necessary to prevent possible residual or metastasis of esophageal squamous carcinomas based on the lymphatic drainage pathway of esophageal carcinomas demonstrated by carbon nanoparticle labeling.

## Introduction

As one of the commonest malignancies, esophageal carcinoma ranks sixth among the commonest causes of cancer-related mortality across the globe, and the incidence is still rapidly on the rise [1, 2] with a poor 5-year survival rate of 10–15% despite multiple approaches of treatment [3]. Radical esophagectomy is the optimal curative choice for resectable esophageal carcinomas, and postoperative prognoses for esophageal carcinomas are determined by the range of the primary cancerous lesion and lymphatic metastasis. Lymph node status is one particular strong prognostic index for survival and recurrence after esophagectomy [4–7]. However, in spite of the clear prognostic implications and recommendations in clinical guidelines, no consensus exists with regard to the therapeutic significance of extensive lymphadenectomy. Some surgeons adopt limited resection strategies, like limited two-field nodal dissection with transthoracic or transhiatal resection, whereas others favor three-field lymph node dissection which may possibly improve long-term survival after esophagectomy [8–11]. A systematic review and meta-analysis has suggested that more radical lymphadenectomy to increase the lymph node yield in esophagectomy is significantly associated with improved disease-free and overall survivals in both Eastern and Western populations [7]. Extended lymphadenectomy may increase physical injury to the patient, especially in open esophagectomy; however, the mortality associated with this approach has not been demonstrated to be increased [7, 12].

For intramucosal and submucosal tumors of the esophagus, endoscopic mucosal resection (EMR) [13, 14], endoscopic submucosal dissection (ESD) [15, 16], and endoscopic full-thickness resection [17–19] have been applied across the world. However, these local surgical procedures entail no lymph node dissection and potentially ignore residual cancer cells or metastasis in the lymphatic draining system. Lymph node spread exists in intramucosal and submucosal esophageal cancers. Approximately 1.93% (95% CI 1.19–2.66%) of T1a intramucosal cancer have positive lymph nodes [20], whereas submucosal invasion (T1b) is associated with a significantly increased risk of lymph node metastasis, with a reported rate of metastasis of 22% in sm1 [21], 23% in sm2 [22], and 69–78% in sm3 [21, 22]. Other researchers have also confirmed a risk of lymph node metastasis of 1.7% for T1a (*P* < 0.001) and 8.6% for T1b (*P* = 0.001) of well- or moderately differentiated esophageal tumors [23]. The left gastric artery lymph nodes are the last stop of regional lymph node metastasis of esophageal carcinomas, and further metastases to the celiac lymph nodes may indicate advanced esophageal carcinoma (stage IV) and M1 stage in the TNM classification. However, is it necessary to dissect the left gastric artery lymph nodes when performing radical esophagectomy for esophageal cancers, especially early-stage cancers, in these patients? Currently, there is no clear answer to this question. With increasing application of endoscopic surgery for patients with esophageal cancers [24–29], this study was to investigate the lymph node metastasis pattern and the significance of dissecting the left gastric artery lymph nodes in en bloc mesoesophageal esophagectomy for esophageal squamous cell cancers based on the lymphatic drainage to the left gastric artery lymph nodes revealed by carbon nanoparticle labeling. Carbon nanoparticles have a mean diameter of 150 nm and can pass through the lymph capillaries to enter ultimately lymph nodes, thus serving as a lymph node tracer during surgeries for thyroid cancer, breast cancer, gastric cancer, and colorectal cancer [30–34]. Labeling of lymph nodes in cancer surgery can facilitate recognition of the range of lymph drainage and complete removal of possible lymph node metastases. The safety of the carbon nanoparticles has been proven by animal experiments [35]. In our study, carbon nanoparticles were injected under thoracoscopy into the submucosa of the upper thoracic esophagus to label the relevant esophageal lymphatic draining system for radical dissection of relevant lymph nodes.

## Materials and methods

This study was approved by the ethics committee of our hospital, and all patients had given the signed informed consent to participate. From January 2017 to March 2021, patients who experienced en bloc thoracolaparoscopic esophagectomy in our hospital were enrolled, with the inclusion criteria of patients of esophageal cancer diagnosed with endoscopic ultrasound examination and biopsy, treated with en bloc esophagectomy and with R0 resection, invasion of adjacent structures (T4), and no distal metastasis confirmed by computed tomography of the chest and abdomen (T4N3M0). The exclusion criteria were patients with extensive metastases confirmed by intraoperative endoscopic exploration, radiotherapy, or neoadjuvant chemotherapy before surgery.

### Surgical technique

The procedure of en bloc esophageal esophagectomy was performed under general anesthesia with tracheal intubation. The total thoracolaparoscopic esophagectomy procedure had three steps: thoracic, abdominal, and cervical operation.

**Fig. 1:**
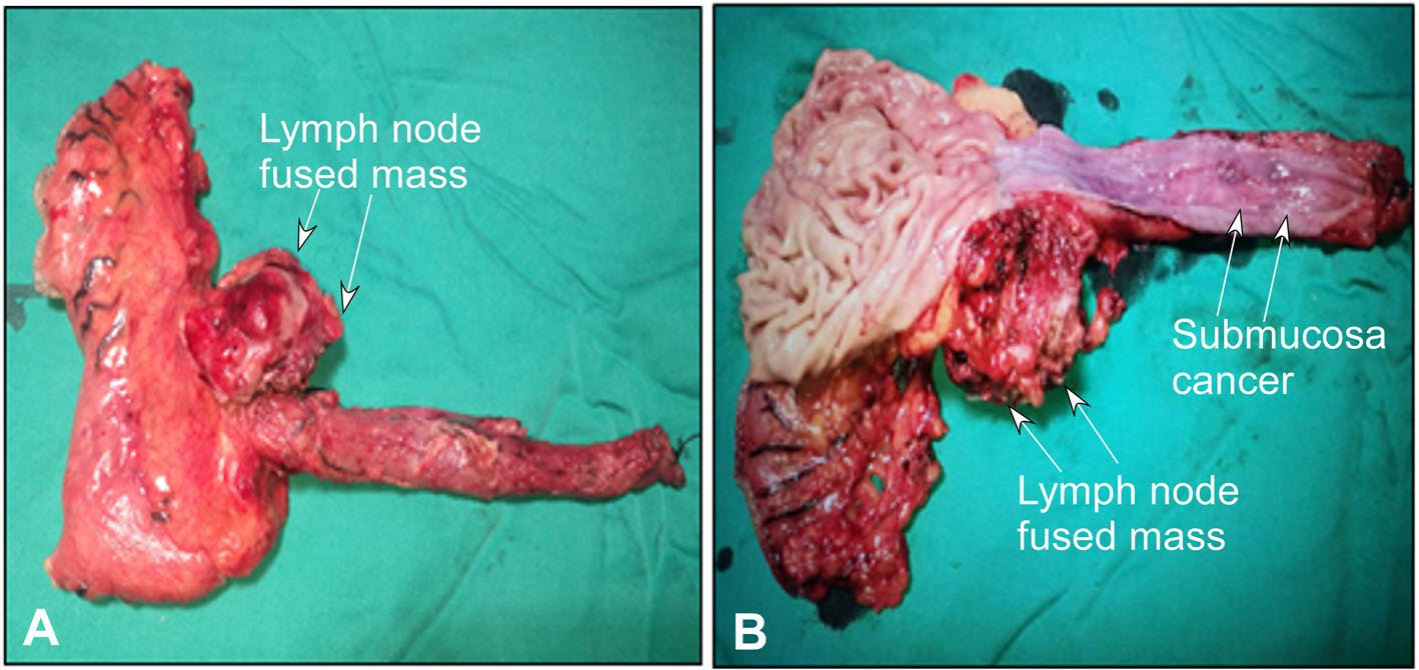
Lymph nodes were dyed with carbon nanoparticles injected into the upper esophagus. **A** Carbon nanoparticle suspension was injected into the submucosa of upper thoracic esophagus. **B** The injection site was shown. **C** The left gastric lymph nodes were labeled with the carbon nanoparticles. **D** A left gastric lymph node was labeled by the nanoparticles. **E** A celiac lymph node was not labeled. **F** The subcarinal lymph node was black-dyed before injection of carbon nanoparticles. **G** The left gastric lymph node was labeled by the carbon nanoparticles (**H**–**E** × 40). **H** The celiac lymph node was not labeled. **I** Deposition of black substances (arrow) was shown in the subcarinal lymph node (**H**–**E** × 40) before injection of the nanoparticles

The thoracoscopic surgery was performed with the patient in the prone position, with one 10-mm Trocar placed in the sixth intercostal space between the right axillary midline and posterior axillary line as an observation port, a 12-mm trocar introduced in the eighth intercostal space under the right scapula as the main operating hole, and a 5-mm trocar placed in the fifth intercostal space at the right scapular line as the second operating hole. Thoracic esophagus was dissociated along the fascial fusion space level up to the upper mediastinal neck and down to the esophageal hiatus. Carbon nanoparticles suspension (2 ml, Chongqing Lummy Pharmaceutical Co., Chongqing, China) was injected into the submucosa of the upper esophagus under endoscopy (Fig. 1). The left and right recurrent laryngeal nerves were mobilized, and the lymph nodes of the recurrent laryngeal nerve and vena cava were dissected.

In the laparoscopic step with five trocars, the patient was changed to the supine position, and laparoscopic mobilization of the stomach and reconstruction of gastric tube were performed. After the left gastric lymph nodes in the left gastric mesentery were labeled by the carbon nanoparticles (Fig. 1D), the esophagus, stomach, left gastric mesentery, blood vessels, lymph nodes, and peripheral connective tissues were dissected en bloc (Fig. 1E).

In the cervical operation, an oblique incision about 5 cm long was made on the left neck along the anterior edge of the sternocleidomastoid muscle. The periesophageal tissues were bluntly separated, and the cervical lymph nodes were dissected. The esophagus was cut off for anastomosis with the gastric tube which was pulled through the esophageal bed upwards for anastomosis end to end with the proximal esophagus. The neck, thoracic, and abdominal wounds were sutured after placement of draining tubes.

### Parameters observed

The clinical data of analysis included age, sex, smoking history, alcohol abuse, hypertension, hyperlipidemia, diabetes mellitus, cancer location and length, cancer differentiation degree, and pathological types. After the procedure, lymph nodes at the neck, subcarina, paraesophagus, cardia, and left gastric artery were dissected and sent for pathological test. The patients were divided into two groups based on positive metastasis of the left gastric artery lymph nodes: positive and negative.

### Statistical analysis

The statistical analysis was performed with the SPSS 19.0 (IBM, Chicago, IL, USA). Measurement data in line with the normal distribution were presented as mean ± standard deviation and tested with the two-sample *t* test, and those in line with the skew distribution were presented as median and interquartile range and tested with the rank sum test. Enumeration data were presented as number and percentages and tested with the Chi square test. Univariant and multivariant logistic regression analyses were performed to investigate the association of cancer metastasis with other factors. The significant *P* was set at < 0.05.

## Results

One hundred and seventy-nine patients who underwent en bloc mesoesophageal esophagectomy through thoracolaparoscopy were enrolled, including 75 women and 104 men with a mean body weight of 63.5 ± 18.2 kg and an age range of 45–90 years (mean 62.5 ± 14.4) (Table 1). The left gastric artery lymph nodes were positive in 42 patients (23.5%, positive group) and negative in 137 (76.5%, negative group). No significant (*P* > 0.05) difference existed in age, sex, smoking and alcohol abuse history, hypertension, hyperlipidemia, diabetes mellitus, cancer location and length, differentiation degree, and pathological types between two groups.

**Table 1:** Demography and clinical data (*n*, %)

Variables	Left gastric artery lymph node positive (*n* = 42)	Left gastric artery lymph node negative (*n* = 137)	*P*
Sex (M/F)	25/17	79/58	0.47
Age (years, mean ± SD)	61.5 ± 10.1	63.6 ± 15.4	0.36
Smoking (*n*)	12 (28.6%)	63 (46.0%)	0.28
Alcohol abuse (*n*)	13 (31.0%)	46 (33.6%)	0.38
Hypertension (*n*)	9 (21.4%)	28 (20.4%)	0.56
Diabetes mellitus (*n*)	2 (4.8%)	8 (5.8%)	0.78
Hyperlipidemia (*n*)	8 (19.0%)	15 (10.9%)	0.57
Cancer location (*n*, %)			
Upper segment	6 (14.3)	9 (6.6)	0.16
Middle segment	21 (50)	70 (51.1)	
Lower segment	15 (35.7)	58 (42.3)	
Cancer length (cm)	2.4–9.5 (5.6)	2.2–8.8 (5.3)	0.79
Infiltration depth (*n*, %)			
Tis	2 (4.8)	6 (4.4)	0.02
T1	12 (28.6)	48 (35.0)	
T2	10 (23.8)	33 (24.1)	
T3	6 (14.3)	27 (19.7)	
T4	12 (28.6)	23 (16.8)	
Pathological type			
Squamous cell carcinoma	39 (92.9%)	132 (96.4%)	0.48
Adenocarcinoma	3 (7.1%)	5 (3.6%)	
Tumor differentiated			
Well	3 (7.1%)	7 (5.1%)	0.19
Moderately	26 (61.9%)	87 (63.5%)	
Low	13 (31.0%)	43 (31.4%)	
Other lymph node metastasis			
Paraesophageal nodes	15 (35.7%)	28 (20.4%)	0.31
Subcarinal nodes	8 (19.0%)	8 (5.8%)	
Para-cardia	7 (16.7%)	10 (7.3%)	
TNM classification			
0	0	1 (0.7%)	0.18
I	4 (9.5%)	11 (8.0%)	
II	4 (9.5%)	71 (51.8%)	
III	26 (61.9%)	51 (37.2%)	
IV	8 (19.0%)	3 (2.2%)	

*SD* standard deviation

Carbon nanoparticle injection was performed in 15 patients at the submucosa of the upper thoracic esophagus. The left gastric lymph nodes were labeled, whereas no celiac lymph nodes were labeled by the carbon nanoparticles (Fig. 1). The subcarinal lymph node was black-dyed before carbon nanoparticles injection (Fig. 1). Pathological examination demonstrated undyed celiac lymph nodes, black-dyed left gastric lymph nodes by the carbon nanoparticles, and naturally black-dyed subcarinal lymph nodes (Fig. 1).

Among 179 patients, a total of 4652 lymph nodes were resected, with 26 (range 23–32) lymph nodes per patient. Seventy-three patients had lymph node metastasis, accounting for 40.8% (73/179). Seventeen (9.5%) patients had metastasis to the recurrent laryngeal nerve lymph nodes. The metastasis rate of the upper thoracic esophageal cancer to the cervical lymph nodes was 33.3% which was significantly greater than that at the middle (7.8%) or lower (6.8%) thoracic segment. The metastasis rate of the lower thoracic esophageal cancer to the left gastric artery lymph nodes was 37.0% which was significantly greater than that at the middle (15.4%) or upper (6.7%) thoracic segment (Table 2). The lymph node metastasis rate was significantly (*P* < 0.05) increased with the length of the cancerous lesion, infiltration depth, and poor differentiation (Table 3).

**Table 2:** Metastasis to the cervical and left gastric artery lymph nodes

Tumor location	Number of patients	Cervical metastasis (*n*, %)	Left gastric artery lymph nodes metastasis (*n*, %)
Upper thoracic segment	15	5 (33.3%)	1 (6.7%)
Middle thoracic segment	91	7 (7.8%)	14 (15.4%)
Low thoracic segment	73	5 (6.8%)	27 (37.0%)
Total	179	17 (9.5%)	42 (23.5%)

**Table 3:** Relationship of cancerous length, infiltration depth, and differentiation with lymph node metastasis

Factors	Number of patients (*n*)	Lymph node metastasis (*n*, %)	*χ*^2^	*P*
Length of cancer				
< 3 cm	34	9(26.5%)	11.98	<0.01
3–7 cm	113	54(47.8%)		
> 7 cm	32	19(59.4%)		
Infiltration depth				
Tis	8	0	52.38	< 0.01
T1	60	8(13.3%)		
T2	43	13(30.2%)		
T3	33	21(63.6%)		
T4	35	31(88.6%)		
Differentiation				
Well	10	2(20%)	21.29	< 0.01
Moderately	113	36(31.9%)		
Low	56	35(62.5%)		

Among 179 patients, the metastasis rate to the left gastric artery lymph nodes was 19.9% (27/136) in patients without involvement of paraesophageal lymph nodes, 20.9% (34/163) in patients without involvement of subcarinal nodes, and 21.6% (35/162) in patients without involvement of para-cardial nodes. The metastasis rate to the left gastric lymph nodes was 7.1% in well-, 61.9% in moderately, and 31.0% in low-differentiated cancers.

**Fig. 2:**
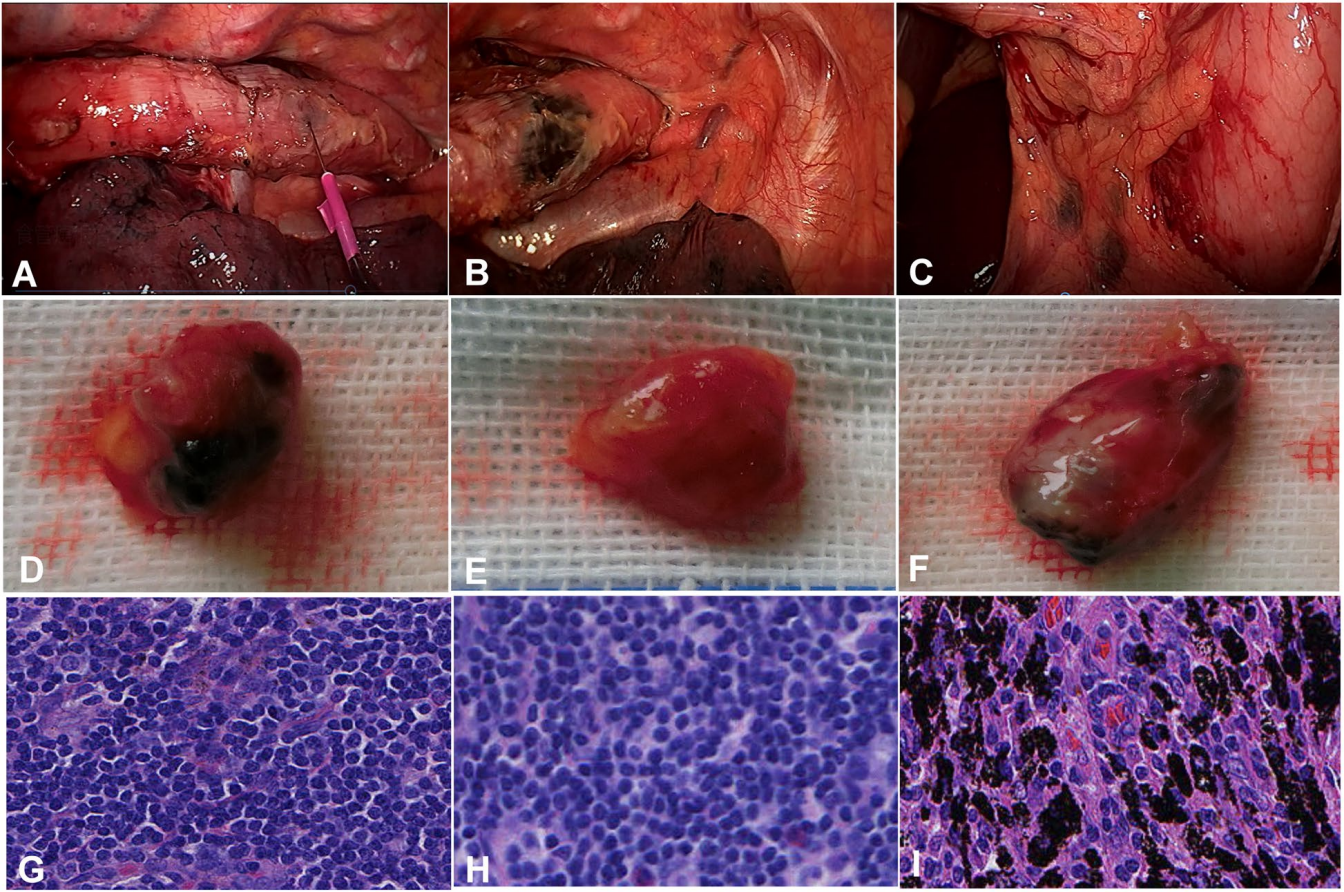
A patient with a submucosal cancer at the middle and lower thoracic esophagus had thoracolaparoscopic en bloc mesoesophageal esophagectomy. **A** The left gastric lymph nodes were enlarged and fused into a 4.3 cm × 5.2 cm mass. **B** The specimen of the esophagus and upper stomach was cut open to show the esophageal submucosa cancer of 1 cm in length

Univariate analysis revealed that the metastasis rate to the left gastric artery lymph nodes was significantly (*P* < 0.05) associated with paraesophageal lymph node metastasis, para-cardial lymph node metastasis, and TNM classification. Multivariate analysis indicated that cancer location (odds ratio 8.32, 95% confidence interval 2.12–32.24) was significantly (*P* < 0.05) associated with metastasis to the left gastric artery lymph nodes, with significantly more metastases to the left gastric artery lymph nodes in cancers at the middle and lower thoracic segments than those in the upper thoracic segment. In one submucosal cancer with 1 cm length at the middle and upper thoracic esophagus, the left gastric lymph nodes were fused into a 4.3 cm × 5.2 cm mass and were completely dissected (Fig. 2).

## Discussion

Through investigating the lymph node metastasis and necessity of dissecting the left gastric artery lymph nodes in en bloc esophagectomy for esophageal squamous carcinomas, it was found that cancer location was significantly (*P* < 0.05) associated with metastasis to left gastric artery lymph nodes, with significantly more metastases to the left gastric artery lymph nodes in cancers at middle and lower thoracic segments than those at the upper thoracic segment. Esophageal cancers even at the early stages can involve the left gastric lymph nodes regardless of differentiation and involvement of other lymph nodes. This indicates the necessity of dissecting left gastric artery lymph nodes in esophagectomy for esophageal carcinomas.

In our study, the mesoesophageal esophagectomy was performed through the thoracolaparoscopic procedure to completely dissect the esophageal squamous carcinoma, mesoesophagus, left gastric artery mesentery, and all the contents within the mesoesophagus and left gastric artery mesentery, including fascial tissues, lymph nodes, blood vessels, adipose tissue, left gastric mesentery, and lymph nodes as an intact package. The left gastric artery mesentery and its contents were all dissected in every patient so as to completely dissect cancer metastasis and prevent residual cancer cells in the left gastric artery mesentery. The left gastric artery lymph nodes are the last stop of regional lymph node metastasis of esophageal carcinomas, and further metastases to the celiac lymph nodes may indicate advanced esophageal carcinoma (stage IV) and M1 stage in the TNM classification. Traditionally, palpable or enlarged left gastric artery lymph nodes were dissected, and this approach will probably result in residual of cancer cells in the lymph nodes without enlargement at this location. If the left gastric artery mesentery and its lymph nodes were all routinely dissected, including all the contents within the mesentery, the spread of cancers within the left gastric artery mesentery and lymph nodes would be prevented, thus delaying cancer development into the M1 stage and improving the survival rate. This is the clinical significance of dissecting all the left gastric artery lymph nodes in all patients with esophageal cancers.

Carbon nanoparticle suspension has been used to trace the lymphatic draining system in cancer surgeries of breast, thyroid, stomach, and colorectum [30–34]. Liu et al. have also applied the carbon nanoparticle suspension in thoracolaparoscopic esophagectomy [31]. The use of carbon nanoparticles can facilitate resection of lymph nodes. The number of lymph nodes dissected was significantly greater in patients labeled with carbon nanoparticle tracers than that in patients without use of carbon nanoparticle tracer [30–34]. In the study investigating the role of nanocarbon lymphatic tracer in thoracolaparoscopic esophagectomy [31], the number of lymph nodes of cancer metastasis in the group of carbon nanoparticle labeling was significantly greater than that in patients without use of the labeling technique. In the study of lymph node mapping with carbon nanoparticles in gastric cancer [36], the average number of harvested lymph nodes in the group with carbon nanoparticle mapping was greater than that in the control group (45.7 ± 14.5 vs. 39.2 ± 11.7) even though the difference was not significant (*P* > 0.05). In the study investigating the effectiveness of carbon nanoparticles for dissection of advanced gastric cancer [37], a significantly higher number of lymph nodes dissected was presented in the group with carbon nanoparticle tracing than in other groups without carbon nanoparticle tracing (34.1 ± 9.8 vs. 25.5 ± 5.5 or 22.6 ± 3.7).

The carbon nanoparticle tracing technique is not applied in all patients in the surgical practice in our hospital. We performed the tracing technique in a certain number of patients to show the lymphatic draining pathway. The use of the tracing technique is to recognize the range and number of lymph nodes to facilitate resection of the lymph nodes [30–34]. Currently, we have applied this technique in fifteen patients to confirm the lymphatic draining pathway to the left gastric artery lymph nodes. If there is only one patient showing the lymphatic draining pathway to the left gastric artery lymph nodes, it means that the esophageal cancer will have lymphatic drainage to this area and that the left gastric artery lymph nodes must be dissected to prevent possible cancer metastasis to this location. Now, fifteen patients have shown this pathway of lymphatic drainage from the esophageal cancer to the left gastric artery lymph nodes. The number of patients may not be sufficient, and in the future, more patients will have to experience this technique for more evidence to dissect the left gastric artery lymph nodes.

In our study, injection of carbon nanoparticles in the submucosa of the upper thoracic segment labeled the left gastric artery lymph nodes, indicating the lymphatic draining direction from the upper thoracic esophageal segment to left gastric artery lymph nodes in the abdominal cavity and the connection of the mesoesophagus to the mesogastrium. The esophagus and stomach originated from the same foregut [38], which is why the mesoesophagus continues downwards with the left gastric mesentery as observed in the endoscopic surgery and indicated by nanoparticle labeling of the left gastric lymph nodes. This constitutes the theoretical basis to resect both the esophageal segment containing the cancer lesion and the upper stomach including the mesogastrium and its contents even if the carcinoma is just an early submucosal lesion in the upper thoracic esophagus. In one patient with an early submucosal cancerous lesion at the thoracic esophagus in our study, the left gastric lymph nodes were fused into a big mass, which indicates the necessity of resecting the upper stomach together with its mesogastrium and left gastric artery lymph nodes besides radical resection of the cancer itself. This may suggest incomplete resection of esophageal submucosal cancer by EMR, ESD, and endoscopic full-thickness resection [13–19, 39–42]. If the esophageal submucosal cancer was just resected locally without removing the upper stomach including the left gastric lymph nodes and mesogastrium, a high proportion of patients with esophageal submucosal carcinomas will have cancer metastasis to the left gastric lymph nodes, resulting in recurrence. It has been revealed that in patients with esophageal cancer limited within the submucosal layer, metastases to lymph nodes were more frequent in the upper mediastinum and perigastric area than in the middle or lower mediastinum [43]. Once the cancer invaded or penetrated the muscular layer, metastasis to lymph nodes dramatically increased in the middle and lower mediastinum even though it was still less frequent than those in the upper mediastinum and the perigastric area.

Injection of carbon nanoparticles in the upper thoracic esophageal segment did not label the celiac lymph nodes, which may indicate that the celiac trunk lymph nodes are not located within the gastric mesentery just like those of the left gastric artery. This also suggests that metastasis is firstly spread to lymph nodes within the mesoesophagus and gastric mesentery before being spread to lymph nodes in other mesenteries. Involvement of the celiac lymph nodes may be a metastatic disease rather than a regional disease as stated in the latest American Joint Committee on Cancer (AJCC) staging edition, which designates involvement of celiac lymph nodes as a regional nodal (N) disease [44].

In our study, the metastasis rate of the upper thoracic esophageal cancer to the cervical lymph nodes was 33.3% which was significantly greater than that at the middle (7.8%) or lower (6.8%) thoracic segment, whereas the metastasis rate of the lower thoracic esophageal cancer to the left gastric artery lymph nodes was 37.0% which was significantly greater than that at the middle (15.4%) or upper (6.7%) thoracic segment. This indicates the bidirectional pattern of esophageal lymph drainage: upwards and downwards. In patients with advanced thoracic esophageal cancer, metastasis may be spread to lymph nodes in the supraclavicular area; upper, middle, and lower mediastinal zones; and perigastric and celiac areas regardless of the cancer location [45]. The abundant lymph draining channels in the esophageal lamina propria mucosa and submucosa are well known, and the submucosal lymphatics primarily drain longitudinally directly to the proximal and distal nodes [46]. Superficial cancers (pT1) can enter into the abundant lymph-capillary plexus in the esophageal lamina propria mucosa and submucosa and spread to distal lymph nodes along with the lymphatic drainage. The bidirectional lymphatic spread pattern of esophageal cancers necessitates three-field radical dissection of lymphatic nodes including the left gastric artery lymph nodes.

In our study, 42 patients with metastasis to the left gastric artery lymph nodes had involvement of paraesophageal nodes in 35.7%, subcarinal nodes in 19.0%, and para-cardial nodes in 16.7%. However, in 137 patients without involvement of the left gastric artery lymph nodes, the metastasis rate was 20.4% to the paraesophageal nodes, 5.8% to the subcarinal nodes, and 7.3% to the para-cardinal nodes. Among 179 patients, the metastasis rate to the left gastric artery lymph nodes was 19.9% (27/136) in patients without involvement of paraesophageal lymph nodes, 20.9% (34/163) in patients without involvement of subcarinal nodes, and 21.6% (35/162) in patients without involvement of para-cardial nodes. This indicates that involvement of the paraesophageal, subcarinal, or para-cardial nodes cannot be used as a reference to dissect the left gastric artery lymph nodes. The metastasis rate to the left gastric lymph nodes was 7.1% in well-, 61.9% in moderately, and 31.0% in low-differentiated cancers, which suggests that the differentiation of the cancer cannot be used as a standard to dissect the left gastric lymph nodes, either.

Some limitations existed in this study, including single-center study, retrospective nature, Chinese patients only, and short follow-up duration, which may all affect the explanation of the outcomes. Moreover, the number of patients experiencing carbon nanoparticle labeling was not sufficient enough, and large numbers of patients with carbon nanoparticle labeling had to be enrolled for more evidence to trace the lymphatic draining pathway from the esophageal cancer to the left gastric artery lymph nodes. Future studies will have to solve these issues to get better results.

In conclusion, certain patterns exist in lymph node metastasis of esophageal cancers, and when performing radical esophagectomy of esophageal cancers, dissection of the left gastric artery lymph nodes is necessary to prevent possible residual and metastasis of esophageal cancers based on the lymphatic drainage pathway of esophageal squamous carcinomas demonstrated by carbon nanoparticle labeling.

## Data Availability

From the corresponding author on reasonable requirement

## References

[1] Jemal A, Bray F, Center MM, Ferlay J, Ward E, Forman D (2011). Global cancer statistics. CA Cancer J Clin.

[2] Mao WM, Zheng WH, Ling ZQ (2011). Epidemiologic risk factors for esophageal cancer development. Asian Pac J Cancer Prev..

[3] Izon AS, Jose P, Hayden JD, Grabsch HI (2013). Significant variation of resected meso-esophageal tissue volume in two-stage subtotal esophagectomy specimens: a retrospective morphometric study. Ann Surg Oncol..

[4] Hofstetter W, Correa AM, Bekele N, Ajani JA, Phan A, Komaki RR (2007). Proposed modification of nodal status in ajcc esophageal cancer staging system. Ann Thorac Surg..

[5] Mariette C, Piessen G, Briez N, Triboulet JP (2008). The number of metastatic lymph nodes and the ratio between metastatic and examined lymph nodes are independent prognostic factors in esophageal cancer regardless of neoadjuvant chemoradiation or lymphadenectomy extent. Ann Surg..

[6] Rice TW, Chen LQ, Hofstetter WL, Smithers BM, Rusch VW, Wijnhoven BP (2016). Worldwide esophageal cancer collaboration: pathologic staging data. Dis Esophagus..

[7] Visser E, Markar SR, Ruurda JP, Hanna GB, van Hillegersberg R (2019). Prognostic value of lymph node yield on overall survival in esophageal cancer patients: a systematic review and meta-analysis. Ann Surg..

[8] Boshier PR, Anderson O, Hanna GB (2011). Transthoracic versus transhiatal esophagectomy for the treatment of esophagogastric cancer: a meta-analysis. Ann Surg..

[9] Colvin H, Dunning J, Khan OA (2011). Transthoracic versus transhiatal esophagectomy for distal esophageal cancer: which is superior?. Interact Cardiovasc Thorac Surg..

[10] Takemura M, Hori T, Fujiwara Y (2013). Clinical outcomes and prognosis after thoracoscopic esophagectomy with two-field lymph node dissection for lower thoracic esophageal cancer. Anticancer Res..

[11] Tsurumaru M, Kajiyama Y, Udagawa H, Akiyama H (2001). Outcomes of extended lymph node dissection for squamous cell carcinoma of the thoracic esophagus. Ann Thorac Cardiovasc Surg..

[12] Hanna GB, Boshier PR, Knaggs A, Goldin R, Sasako M (2012). Improving outcomes after gastroesophageal cancer resection: can japanese results be reproduced in western centers?. Arch Surg..

[13] Noordzij IC, Curvers WL, Schoon EJ (2019). Endoscopic resection for early esophageal carcinoma. J Thorac Dis..

[14] Seewald S, Ang TL, Pouw RE, Bannwart F, Bergman JJ (2018). Management of early-stage adenocarcinoma of the esophagus: endoscopic mucosal resection and endoscopic submucosal dissection. Dig Dis Sci..

[15] Lee IL, Lin PY, Tung SY, Shen CH, Wei KL, Wu CS (2006). Endoscopic submucosal dissection for the treatment of intraluminal gastric subepithelial tumors originating from the muscularis propria layer. Endoscopy..

[16] Shi Q, Zhong YS, Yao LQ, Zhou PH, Xu MD, Wang P (2011). Endoscopic submucosal dissection for treatment of esophageal submucosal tumors originating from the muscularis propria layer. Gastrointest Endosc..

[17] Hiki N, Yamamoto Y, Fukunaga T, Yamaguchi T, Nunobe S, Tokunaga M (2008). Laparoscopic and endoscopic cooperative surgery for gastrointestinal stromal tumor dissection. Surg Endosc..

[18] Schlag C, Wilhelm D, von Delius S, Feussner H, Meining A (2013). Endoresect study: endoscopic full-thickness resection of gastric subepithelial tumors. Endoscopy..

[19] Tang X, Ren Y, Huang S, Gao Q, Zhou J, Wei Z (2017). Endoscopic submucosal tunnel dissection for upper gastrointestinal submucosal tumors originating from the muscularis propria layer: A single-center study. Gut Liver..

[20] Dunbar KB, Spechler SJ (2012). The risk of lymph-node metastases in patients with high-grade dysplasia or intramucosal carcinoma in Barrett's esophagus: a systematic review. Am J Gastroenterol..

[21] Bollschweiler E, Baldus SE, Schroder W, Prenzel K, Gutschow C, Schneider PM (2006). High rate of lymph-node metastasis in submucosal esophageal squamous-cell carcinomas and adenocarcinomas. Endoscopy..

[22] Buskens CJ, Westerterp M, Lagarde SM, Bergman JJ, ten Kate FJ, van Lanschot JJ (2004). Prediction of appropriateness of local endoscopic treatment for high-grade dysplasia and early adenocarcinoma by eus and histopathologic features. Gastrointest Endosc..

[23] Gamboa AM, Kim S, Force SD, Staley CA, Woods KE, Kooby DA (2016). Treatment allocation in patients with early-stage esophageal adenocarcinoma: prevalence and predictors of lymph node involvement. Cancer..

[24] Cuesta MA, Weijs TJ, Bleys RL, van Hillegersberg R, van Berge Henegouwen MI, Gisbertz SS (2015). A new concept of the anatomy of the thoracic oesophagus: the meso-oesophagus. Observational study during thoracoscopic esophagectomy. Surg Endosc..

[25] Guo W, Ma X, Yang S, Zhu X, Qin W, Xiang J (2016). Combined thoracoscopic-laparoscopic esophagectomy versus open esophagectomy: a meta-analysis of outcomes. Surg Endosc..

[26] Maas KW, Biere SS, Scheepers JJ, Gisbertz SS, van-der-Peet DL, Cuesta MA (2012). Laparoscopic versus open transhiatal esophagectomy for distal and junction cancer. Rev Esp Enferm Dig..

[27] Merritt RE, Kneuertz PJ, D'Souza DM, Perry KA (2019). Total laparoscopic and thoracoscopic ivor lewis esophagectomy after neoadjuvant chemoradiation with minimal overall and anastomotic complications. J Cardiothorac Surg..

[28] Murakami M, Otsuka K, Goto S, Ariyoshi T, Yamashita T, Aoki T (2017). Thoracoscopic and hand assisted laparoscopic esophagectomy with radical lymph node dissection for esophageal squamous cell carcinoma in the left lateral decubitus position: a single center retrospective analysis of 654 patients. BMC Cancer..

[29] Wan J, Che Y, Kang N, Zhang R (2016). Surgical method, postoperative complications, and gastrointestinal motility of thoraco-laparoscopy 3-field esophagectomy in treatment of esophageal cancer. Med Sci Monit..

[30] Li Z, Ao S, Bu Z, Wu A, Wu X, Shan F (2016). Clinical study of harvesting lymph nodes with carbon nanoparticles in advanced gastric cancer: a prospective randomized trial. World J Surg Oncol..

[31] Liu L, Zhu J, Lu C, Zhou J (2017). The role of nanocarbon lymphatic tracer in thoraco-laparoscopic esophagectomy. Minerva Chir..

[32] Wang LY, Li JH, Zhou X, Zheng QC, Cheng X (2017). Clinical application of carbon nanoparticles in curative resection for colorectal carcinoma. Onco Targets Ther..

[33] Zhang L, Huang Y, Yang C, Zhu T, Lin Y, Gao H (2018). Application of a carbon nanoparticle suspension for sentinel lymph node mapping in patients with early breast cancer: a retrospective cohort study. World J Surg Oncol..

[34] Zhao WJ, Luo H, Zhou YM, Gou ZH, Wang B, Zhu JQ (2017). Preoperative ultrasound-guided carbon nanoparticles localization for metastatic lymph nodes in papillary thyroid carcinoma during reoperation: a retrospective cohort study. Medicine (Baltimore).

[35] Xie P, Yang ST, He T, Yang S, Tang XH. Bioaccumulation and toxicity of carbon nanoparticles suspension injection in intravenously exposed mice. Int J Mol Sci. 2017;18(12):2562. 10.3390/ijms18122562. 10.3390/ijms18122562 PMC5751165 29186019

[36] Wang H, Chen MM, Zhu GS, Ma MG, Du HS, Long YP (2016). Lymph node mapping with carbon nanoparticles and the risk factors of lymph node metastasis in gastric cancer. J Huazhong Univ Sci Technolog Med Sci..

[37] Mu G, Huang Y, Wei C, Chen Z, Wu X, Qin X (2020). Para-aortic lymph node tracing and dissection in advanced gastric cancer: effectiveness of carbon nanoparticles injection through the no. 12b lymph node. J Cancer Res Ther..

[38] Tachimori Y (2014). Total mesoesophageal esophagectomy. Chin Med J (Engl).

[39] Chai N, Du C, Gao Y, Niu X, Zhai Y, Linghu E (2018). Comparison between submucosal tunneling endoscopic resection and video-assisted thoracoscopic enucleation for esophageal submucosal tumors originating from the muscularis propria layer: a randomized controlled trial. Surg Endosc..

[40] Peng W, Tan S, Huang S, Ren Y, Li H, Peng Y (2019). Efficacy and safety of submucosal tunneling endoscopic resection for upper gastrointestinal submucosal tumors with more than 1-year' follow-up: a systematic review and meta-analysis. Scand J Gastroenterol..

[41] Wadhwa V, Franco FX, Erim T (2020). Submucosal tunneling endoscopic resection. Surg Clin North Am..

[42] Zhang M, Wu S, Xu H (2019). Comparison between submucosal tunneling endoscopic resection (ster) and other resection modules for esophageal muscularis propria tumors: a retrospective study. Med Sci Monit..

[43] Tachimori Y, Nagai Y, Kanamori N, Hokamura N, Igaki H (2011). Pattern of lymph node metastases of esophageal squamous cell carcinoma based on the anatomical lymphatic drainage system. Dis Esophagus.

[44] Wen JCD, Zhao T, Chen J, Zhao Y, Liu D, Wang W, Xu X, Fan M, Chen C, Chen Y (2019). Should the clinical significance of supraclavicular and celiac lymph node metastasis in thoracic esophageal cancer be reevaluated. Thorac Cancer..

[45] Kumakura YYT, Yoshida T, Hara K, Sakai M, Sohda M, Miyazaki T, Yokoo H, Handa T, Oyama T, Yorifuji H, Kuwano H (2018). Elucidation of the anatomical mechanism of nodal skip metastasis in superficial thoracic esophageal squamous cell carcinoma. Ann Surg Oncol..

[46] Tachimori Y (2017). Pattern of lymph node metastases of squamous cell esophageal cancer based on the anatomical lymphatic drainage system: efficacy of lymph node dissection according to tumor location. J Thorac Dis.

